# Maturational delay in ADHD: evidence from CPT

**DOI:** 10.3389/fnhum.2013.00691

**Published:** 2013-10-25

**Authors:** Itai Berger, Ortal Slobodin, Merav Aboud, Julia Melamed, Hanoch Cassuto

**Affiliations:** ^1^Pediatric Division, The Neuro-Cognitive Center, Hadassah-Hebrew University Medical CenterJerusalem, Israel; ^2^Faculty of Medicine, Hebrew University - Hadassah Medical CenterJerusalem, Israel; ^3^Leumit HMO, Pediatric NeurologyJerusalem, Israel

**Keywords:** ADHD, CPT, symptoms, maturation, delay, diagnosis

## Abstract

While data from behavioral, neuropsychological, and brain studies suggested that Attention-Deficit/Hyperactivity Disorder (ADHD) is related to a developmental lag that reduces with age, other studies have proposed that ADHD represents a deviant brain function. The present study used a cross-sectional approach to examine whether ADHD children show a developmental delay in cognitive performance measured by continuous performance test (CPT). We thus, compared six age groups of ADHD children (*N* = 559) and their unaffected peers (*N* = 365), aged 6–11, in four parameters of MOXO-CPT performance: Attention, Timing, Hyperactivity and Impulsivity. Results have shown that despite improvement in CPT performance with age, ADHD children continued to demonstrate impaired performance as compared to controls. In most parameters, CPT performance of ADHD children matched that of 1–3 years younger normal controls, with a delay most prominent in older children. However, in the Hyperactivity parameter, ADHD children's performance resembled that of much younger healthy children, with almost no evidence for a developmental catch up. This study suggests that while some cognitive functions develop slower but normally, other functions (e.g., inhibitory control) show a different trajectory.

## Introduction

Attention-deficit hyperactivity disorder (ADHD) is the most common neurobehavioral disorders of childhood, characterized by inattention, impulsivity and hyperactivity. Using the DSM-IV criteria [American Psychiatric Association (APA), 2000], prevalence rates in the United States range from 7.4 to 9.9% (Barkley, 2006). There is growing evidence that ADHD has important developmental aspects and its symptoms change considerably over time (Greenberg and Waldman, 1993; Hart et al., 1995; Faraone et al., 2006). Leading researchers (Barkley, 1990, 1997; Gillberg, 2010; Sonuga-Barke and Halperin, 2010) have long argued that ADHD is a “developmental disorder” with early onset and that deficits in inhibition appear in early childhood leading to a cascade of other problems in self-regulation, encompassed under the rubric of executive functioning.

Many children with ADHD have been described as having co-morbid developmental problems in motor coordination, language, behavior, sleep, and mood (Hartsough and Lambert, 1985; Gillberg and Kadesjo, 2003; Kalff et al., 2003; Gillberg, 2010)

Although ADHD symptoms often persist over time (Greydanus et al., 2007), maturation has a significant positive effect on ADHD symptoms in many children (Faraone et al., 2000). These observations have given rise to the hypothesis that ADHD is related to a delay rather than a deviance of normal brain development (Kinsbourne, 1973; Steffensson et al., 1999; El-Sayed, 2002).

According to the “maturational lag” model, ADHD children have neurodevelopment profiles representative of healthy children at younger ages (Kinsbourne, 1973). As a child with ADHD gets older and “catches up” the developmental lag, the symptoms of ADHD might lessen. This model was initially based on the behavioral observation that children with ADHD often behave as younger children, who naturally have lesser ability to sustain attention, display impulse control, and sit still for a long time period.

In support of this model, two longitude studies using computational neuroanatomic techniques demonstrated that children with ADHD follow a similar sequential pattern of cortical development, yet were delayed by as much as 2–3 years, depending upon the specific cortical region (Shaw et al., 2007, 2012). Shaw et al. (2007) used the peak of cortical thickness as delineating a phase of childhood increase followed by adolescent decrease in cortical thickness. Results showed that while the peak in cortical thickness was attained in the cerebrum around 7 years in typically developing children, in children with ADHD, peak cortical thickness was reached around 10 years, with the delay most prominent in lateral prefrontal cortex. In the second longitudinal study, delayed brain maturation (of ~2 years) in ADHD children was reported in the cortical surface area (Shaw et al., 2012). The authors concluded the congruent delay in both cortical thickness and surface area in ADHD represents a global perturbation in the mechanisms that guide cortical maturation.

Indirect neurobiological support to the maturation-lag model comes from cross-sectional structural imaging studies which yielded reduced size in cortico-striatal brain regions that are known to develop late in adolescence (Krain and Castellanos, 2006). Additionally, research of brain activity demonstrated underactivation in those regions where function develops linearly with age between childhood and adulthood (Krain and Castellanos, 2006; Rubia et al., 2006; Smith et al., 2006). Electroencephalography (EEG) studies have documented increased slow wave activity (mostly theta) (Lazzaro et al., 2001; Clarke et al., 2002; El-Sayed et al., 2002; Yordanova et al., 2009) in preadolescent and adolescents with ADHD compared with normal controls. This finding has been interpreted as different arousal level in children with ADHD, which could be due to a delay in functional cortical maturation (Mann et al., 1992).

Further evidence for the maturational lag model was found in neuropsychological functioning of ADHD children. ADHD children showed later development of executive functions, such as inhibitory self-control, attention, and temporal foresight, which are mainly dependent on circuits in the frontal lobes (Barkley, 1997; Kalff et al., 2003; Rubia et al., 2007). For example, Shue and Douglas (1992) have demonstrated that on tests sensitive to frontal lobe functions (but not temporal lobe) ADHD children lagged 3–4 years behind their healthy peers. However, ADHD deficits in neuropsychological performance were not necessarily related to brain developmental delay. In order to test whether ADHD is related to a maturational lag in brain development, Doehnert et al. (2010) examined CPT performance and ERP (event related potentials) markers of attention and inhibitory control deficits in ADHD and non-ADHD children in three time points. Although CPT performance was consistent with the developmental lag model, ERP data did not support the developmental lag hypothesis for attentional dysfunction in ADHD. Results showed that ADHD effects may mimic age effects at the level of behavior or performance but these effects were unrelated to patterns of neural activation. Additional studies using ERP (Johnstone et al., 2001; Smith et al., 2004), Magnetic Resonance Imaging (MRI) (Castellanos et al., 2000) and functional Magnetic Resonance Imaging (fMRI) (Mostofsky et al., 2006; Zhu et al., 2008) indicated that ADHD deficits shared little in common with the pattern of brain activity seen in younger control children, which suggests that ADHD children may have a deviant brain function rather than a maturation delay.

While ADHD symptoms and neuropsychological dysfunction are correlated (Nigg, 2005; Seidman, 2006) it is still unclear to which degree neuropsychological functioning parallels the attenuation of ADHD symptoms over time. Evidence suggests that children with ADHD continued to exhibit impaired neuropsychological functioning despite clinical improvement of ADHD symptoms (Fischer et al., 2005; Halperin et al., 2008; Hinshaw et al., 2007). For example, Hinshaw et al. (2007) found that commission errors in the Conners' CPT were not related to ADHD diagnostic status over a 5 year period (persisters and remitters did not differ on this outcome at follow up). In contrast, other studies (Fischer et al., 2005; Halperin et al., 2008) reported that persisters, but not remitters were significantly differentiated from controls on commission errors on an identical pairs CPT task. To explain the association between behavioral and neuropsychological functioning of ADHD across the life-span, Halperin and Schulz (2006) argued that ADHD is caused by non-cortical neural dysfunction that is present early in ontogeny, remains relatively static throughout life, and is not associated with the reduction of symptoms typically seen over development. Age- related symptom reduction is attributed to prefrontally-mediated executive functions compensating for more primary and enduring subcortical deficits. According to this model, neuropsychological deficits on task measuring effortful controlled processing (e.g., commission errors on a go/no-go task) should decrease with maturation paralleling the reduction of ADHD symptomatology. On the other hand, neuropsychological deficits on tasks measuring automatic and less conscious control (e.g., reaction time variability) tend to persist over time remaining unrelated to ADHD symptom presentation.

Most of the longitudinal studies addressing ADHD manifestations over time examined ADHD symptoms dichotomously (i.e., either the patient meets ADHD criteria or not) (Vaughn et al., 2011). Because the use of diagnostic stability is related to the definition of remission, it changes significantly between studies (Biederman et al., 2000; Spencer et al., 2002; Faraone et al., 2006). For instance, when ADHD samples included only those who met full diagnostic criteria for ADHD the rate of persistence was ~15% at age of 25 years. However, when partial remission was also included, almost two thirds of ADHD cases suffered from significant clinical impairments in adulthood (Faraone et al., 2006). Another problem with many longitudinal studies is that they use long follow up that may be insensitive to smaller changes in performance. Thus, Vaughn et al. (2011) highlightened the need to include more frequent assessments over a longer period of time, to fully map the likely non-linear developmental trajectories.

The present study used a cross-sectional approach in order to examine whether ADHD children show a developmental delay in CPT performance that mirrors the delayed maturation documented in brain development studies. We hypothesized that ADHD children will perform worse than normal controls in CPT and that their performance would consistently match that of younger typically developed children. We thus, compared six age groups of ADHD children and their unaffected peers (6–11 years) in four parameters of CPT performance to determine whether the disorder is characterized by a delay in cognitive development.

## Materials and methods

### Participants

Participants in this study were 924 children aged 6–11 years, of them 539 boys and 385 girls. The ADHD group included 559 children diagnosed with ADHD and the control group included 365 children without ADHD. The children were divided into six age categories (6–11 years). For example, the category of “8 years” included children who were equal or older than 8 years old, but younger than 9 years old. Background variables are presented in Table 1. In the majority of age groups, the ADHD and control groups did not differ in age or gender distributions. In the group of 10 years, the control group were slightly older than the ADHD group (mean age of 10.60 vs., 10.45 years, respectively). The ADHD group included more boys relatively to the control group at ages 6 and 7.

**Table 1:** **participants' background variables**.

**Age category**		**ADHD (***N*** = **559**)**		**Control (***N*** = **365**)**		**Difference**
6	N	107		53		
	Male	76 (71.03%)		27 (50.94%)		χ^2^_(1,*N* = 160)_ = 6.23^*^
	female	31 (28.97%)		26 (49.06%)		
	Age M (*SD*)	6.53	(0.30)	6.57	(0.27)	*t*_(158)_= −0.88
7	N	111		94		
	Male	73 (65.77%)		39 (41.49%)		χ^2^_(1,*N* = 205)_ = 12.20^***^
	female	38 (34.23%)		55 (58.51%)		
	Age M (*SD*)	7.45	0.02	7.46	0.03	*t*_(203)_ = −0.22
8		112		70		
	Male	66 (58.93%)		33 (47.14%)		χ^2^_(1,*N* = 182)_ = 2.41
	female	46 (41.07%)		37 (52.86%)		
	Age M (*SD*)	8.51	0.28	8.45	0.32	*t*_(180)_ = 1.30
9	N	93		57		
	Male	56 (60.22%)		33 (57.89%)		χ^2^_(1,*N* = 150)_ = 0.08
	female	37 (39.78%)		24 (42.11%)		
	Age M (*SD*)	9.51	0.27	9.53	0.28	*t*_(148)_ = −0.31
10	N	77		59		
	Male	47 (61.04%)		32 (54.24%)		χ^2^_(1,*N* = 136)_ = −0.63
	female	30 (38.96%)		27 (45.76%)		
	Age M (*SD*)	10.46	0.31	10.60	0.28	*t*_(134)_ = −2.67^**^
11	N	59		32		
	Male	39 (66.10%)		18 (56.25%)		χ^2^_(1,*N* = 91)_ = 0.86
	female	20 (33.90%)		14 (43.75%)		
	Age M (*SD*)	11.50	0.33	11.39	0.28	*t*_(89)_= 1.47

* p < 0.05;

** p < 0.01;

*** p < 0.001.

Participants in the ADHD group were recruited from children referred to the out-patient paediatric clinics of a Neuro-Cognitive Center, based in a tertiary care university hospital. The children were referred through their paediatrician, general practitioner, teacher, psychologist, or directly by the parents.

Inclusion criteria for participants in the ADHD group were:

1. Each child met the criteria for ADHD according to DSM-IV-TR criteria (APA, 2000), as assessed by a certified paediatric neurologist. The diagnostic procedure included an interview with the child and parents, fulfilment of questionnaires, and medical/neurological examination that confirmed ADHD diagnosis.
2. Each child scored above the standard clinical cut off values for ADHD symptoms on ADHD/DSM-IV Scales (APA, 2000).
3. All children were drug naïve.

Participants in the control group were randomly recruited from pupils in regular classes at primary schools. Inclusion criteria for participants in the control group were:

1. Each child scored below the clinical cut off point for ADHD symptoms on ADHD/DSM-IV Scales (APA, 2000).
2. Absence of academic or behavioral problems, as reported by parents and teachers.

Exclusion criteria were intellectual disability, other chronic condition, chronic use of medications, and other primary psychiatric diagnosis (e.g., depression, anxiety, and psychosis). All participants agreed to participate in the study and their parents gave written informed consent to the study, approved by the Helsinki committee (IRB) of Hadassah-Hebrew University Medical Center (Jerusalem, Israel).

### Measures

#### Measurement of child behavior

The parent and teacher forms of the Conner's ADHD/DSM-IV Scales were used to assess the level of children's ADHD behaviors (Conners, 1997a,b; APA, 2000).

#### The MOXO continuous performance test

This study employed the MOXO-CPT version^1^ (Berger and Goldzweig, 2010), which is a standardized computerized test designed to diagnose ADHD related symptoms. The test included visual and auditory stimuli that serve as distractors.

**Figure 1:**
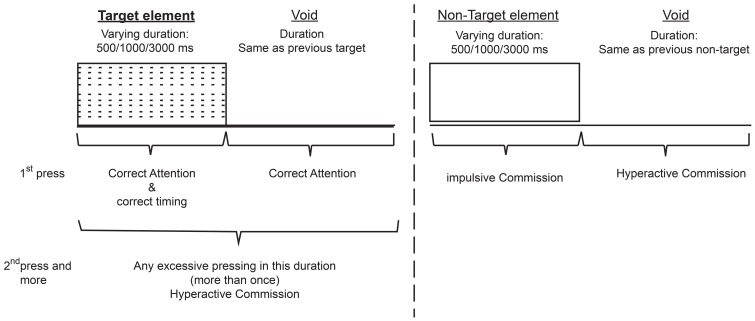
**Definition of the time line (Target and non-target stimuli were presented for 500, 1000, or 3000 ms**. Each stimulus was followed by a void period of the same duration. The stimulus remained on the screen for the full duration regardless the response. Distracting stimuli were not synchronized with target/non-target's onset and could be generated during target/non-target stimulus or during the void period).

The total duration of the test was 15.2 min, and it is composed of eight levels (114.15 s, 53 trials each). In each trial a stimulus (target/non-target) was presented for 500, 1000, or 3000 ms and then followed by a “void” period of the same duration (Figure 1). The stimulus remained on the screen for the full duration no matter if a response was produced. This practice allowed the measuring response timing (whether the response occurred during stimulus presentation or the void period) as well as the accuracy of the response.

In each level 33 target and 20 non-target stimuli were presented. Both target and non-target stimuli were cartoon pictures that do not include any letters. The absence of letters is important given the fact that ADHD patients tend to have learning difficulties e.g., dyslexia, dyscalculia) that may be confound with CPT performance (Seidman et al., 2001). The stimuli were presented sequentially in the middle of a computer screen and the participant was instructed to respond as quickly as possible to target stimuli by pressing the space bar once, and only once. The participant was also instructed not to respond to any other stimuli except the target, and not to press any other key but the space bar.

Test level and distracting stimuli—In order to simulate everyday environment of children, the MOXO-CPT contained distracting stimuli. This feature is unique to this specific CPT. Distractors were short animated video clips containing visual and auditory features which can appear separately or together. This enabled to present three types of distractions that characterize everyday environment: (a) visual distractors (e.g., animated flying bird); (b) auditory distractors (e.g., bird singing); and (c) combination of both visual and auditory distractors (e.g., animated flying bird with the sound of a bird singing).

Overall, six different distractors were included, each of them could appear as pure visual, pure auditory or as a combination of them. Each distractor was presented for a different duration ranging from 3.5–14.8 s, with a fixed interval of 0.5 s between two distractors. Distractors' onset was not synchronized with target/non-target's onset and could be generated during target/non-target stimulus or during the void period. Visual distractors appeared at one of four spatial locations on the sides of the screen: down, up, left or right. Different levels of the MOXO-CPT were characterized by a different set of distractors: levels 1 and 8 did not include any distractors but only target and non-target stimuli, levels 2 and 3 contained pure visual stimuli, levels 4 and 5 contained pure auditory stimuli, and levels 6 and 7 contained a combination of visual and auditory stimuli. The sequence of distracters and their exact position on the display were constant for each level. The burden of the distracting stimuli increased at the odd number levels; in the 2nd, 4th, and 6th level only one distractor was presented at a time, while in the 3rd, 5th, and 7th level two distractors were presented simultaneously.

***Performance indices.*** The MOXO-CPT included four performance indices: attention, Timing, Impulsivity, and Hyperactivity. For detailed description of performance indices see Supplementary A.

***Attention.*** This index corresponded to the number of correct responses (a space bar keystroke in response to a target stimulus) performed during the stimulus presentation or the void period that followed it. This index was considered as a pure measure of sustained attention because it measured correct responses independently of the response time.

***Timing.*** The timing index was the number of correct responses given only during the time in which the target stimulus was present on the screen.

***Impulsivity.*** The impulsivity index was the number of commission responses performed only during the time in which a non-target stimulus was present on the screen.

***Hyperactivity.*** The hyperactivity index was the total number of commission responses that were not coded as impulsive responses (e.g., multiple keystrokes in response to a target stimulus, responses performed in the void period after a non-target stimulus, random key pressing).

### Data analyses

All analyses were conducted with SAS software for Windows version 9.2. First, *T*-tests for independent samples and chi-square tests were used for examining group differences across demographic variables. Second, *T*-tests for independent samples were used to measure the effect of group on CPT indices. Then, each age category of ADHD children was matched to a group of typically developing children which had the closest mean value in the same parameter, by using Cohen's d measure (absolute difference in the mean values of the two groups divided by pooled standard deviation for each age.

## Results

First, differences in CPT performance parameters (Attention, Timing, Hyperactivity, and Impulsivity) between ADHD children and their age-matched healthy peers were examined by two tailed *t*-test analyses for independent samples.

As can be seen in Table 2, in all age groups children with ADHD received significantly lower scores in the Attention and Timing parameters than normal controls. That is, ADHD children were less attended to the stimuli and performed less reactions on accurate time. In age groups 6, 7, and 10 ADHD children produced significantly more hyperactive and impulsive responses as compared to non-ADHD children. Marginally significant differences between the two groups were observed at ages 8 and 11 in hyperactivity responses (*p* = 0.07 and *p* = 0.08, respectively) and at age 9 for impulsivity responses (*p* = 0.06). The rest of the comparisons did not yield significant group differences.

**Table 2:** **Differences between ADHD children and their typically developed peers in MOXO-CPT performance**.

**Age category (Years)**	**MOXO-CPT parameter**	**ADHD (***N*** = **559**)**		**Control (***N*** = **365**)**		***t***	***df***	***p*(2-tailed)**
		**Mean**	**(*SD*)**	**Mean**	**(*SD*)**			
6	N	107		53				
	Attention	211.1	43.75	234	22.24	−3.59	158	<0.001
	Timing	140.8	37.58	157	33.99	−2.64	158	<0.01
	Hyperactive	97.52	141.5	41.71	33.23	2.83	158	<0.01
	Impulsivity	28.23	33.04	15.70	10.79	2.69	158	<0.01
7		111		94				
	Attention	231.8	25.01	246.7	13.75	−5.18	203	<0.001
	Timing	160.2	33.17	177.1	27.18	−3.94	203	<0.001
	Hyperactive	64.23	63.64	38.21	23.23	3.76	203	<0.001
	Impulsivity	19.38	12.16	15.83	9.31	2.31	203	<0.05
8		112		70				
	Attention	242.8	15.10	249.4	14.02	−2.92	180	<0.01
	Timing	175.6	28.71	190.4	26.53	−3.48	180	<0.001
	Hyperactive	52.14	97.50	30.33	27.81	1.82	180	0.07
	Impulsivity	16.5	12.37	14.94	9.30	0.90	180	0.37
		93		57				
9	Attention	242	35.78	253.4	10.48	−2.33	148	<0.05
	Timing	190.3	41.27	205.2	23.32	−2.48	148	<0.05
	Hyperactive	44.57	50.01	32.26	32.01	1.52	148	0.13
	Impulsivity	18.32	11.74	15.11	6.53	1.89	148	0.06
		77		59				
10	Attention	246.5	25.19	255.3	12.55	−2.47	134	<0.05
	Timing	197.5	35.78	217.7	24.23	−3.72	134	<0.001
	Hyperactive	40.19	40.72	24.98	29.70	2.42	134	<0.05
	Impulsivity	16.36	9.73	13.18	7.51	2.08	134	<0.05
		59		32				
11	Attention	250	15.91	258	8.12	−2.66	89	<0.01
	Timing	205.7	29.48	228.3	19.10	−3.91	89	<0.001
	Hyperactive	30.08	36.08	17.47	23.14	1.79	89	0.08
	Impulsivity	14.23	11.09	13.22	7.07	0.47	89	0.64

**Figure 2:**
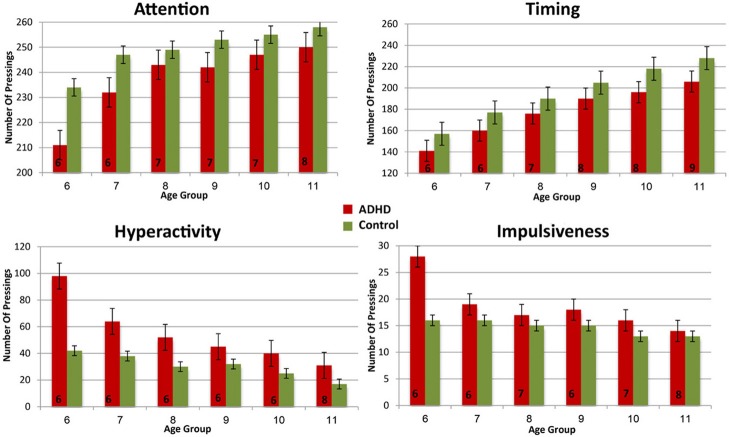
**Performance in four CPT parameters among ADHD children and control group**.

In order to evaluate the developmental trajectories of the attention performance, each age category of ADHD children was matched to a group of typically developing children which had the closest mean value in the same parameter. The matched group was chosen by using Cohen's d measure (absolute difference in the mean values of the two groups divided by pooled standard deviation for each age) (Tables B1–B4, Appendix B). Results are shown in Figure 2. As can be seen in the figures, both ADHD and control groups showed higher scores in Attention and Timing parameters and lower scores in Hyperactivity and Impulsivity with maturation, but the performance of ADHD children matched that of younger healthy controls. In the Attention parameter, the performance of 6–7 years old ADHD children closely resembled the performance of 6 years old typically developing children. Furthermore, the performance of 8–10, and 11 years old ADHD children closely resembled that of a 7 and 8 years old typically developing children, respectively. A very similar pattern was found for the Timing parameter: performance of 6–7 years ADHD children closely resembled the performance of 6 years old typically developing children. The performance of 8, 9–10, and 11 years old ADHD children closely resembled that of 7, 8, and 9 years old typically developing children, respectively. A slightly different, non-linear, pattern was obtained in the Impulsivity parameter, in which 6–7 and 9 years old ADHD children performed as 6 years old non-ADHD children, 8 and 10 ADHD children performed as 7 years old non-ADHD children, and 11 years old ADHD performed as 8 years old non-ADHD. In the Hyperactivity parameter, ADHD children aged 6–10 performed as 6 years old controls, whereas 11 years old ADHD children performed similar to 8 years old children.

In most CPT indices, except Hyperactivity, ADHD children consistently lagged 1–3 years behind their typically developed peers. However, the delay was more prominent in older ages: while at ages 6–8, CPT performance of ADHD children resembled that of 6–7 years old controls, at ages 10-11, ADHD children were more likely to perform as 7–8 years old controls.

## Discussion

This paper examined CPT performance of ADHD and non-ADHD children, in order to determine whether the disorder is characterized by a delayed development of attentional functions. Consistent with previous literature (Drechsler et al., 2005; Doehnert et al., 2010; Vaughn et al., 2011), our results have shown that ADHD children of all ages were significantly more inattentive and performed fewer reactions on accurate timing than the control group. In some age groups (6, 7, and 10 years), children with ADHD also produced significantly more hyperactive and impulsive responses than non-ADHD children, whereas in others (8, 9, and 11 years) only marginal or no group effects were found. This finding indicated that despite improvement in CPT performance, ADHD children continue to demonstrate impaired functioning as compared to healthy controls.

In line with findings from longitudinal studies (Shaw et al., 2007, 2012; Vaughn et al., 2011), our results revealed that ADHD and typically developing children showed a similar sequence of development in their attention capacities, but on a different time. In most CPT parameters, performance of ADHD children, delayed and matched that of 1–3 years younger healthy controls.

This pattern of maturation-lag in CPT performance mirrors the 2–3 delayed maturation of the brain in ADHD children (Shaw et al., 2007, 2012). In this context, the current study suggests that at least part of the difficulties of ADHD children could be explained by developmental delay that improves with time. Nevertheless, cautions should be taken when interpreting maturation lag in CPT performance as directly associated with a parallel lag in brain development. As reported previously, the two domains may not be directly linked (Doehnert et al., 2010). More large scale longitudinal studies of brain structure and function are required to address this point (Sonuga-Barke, 2010).

Inconsistent with Halperin and Schulz's (2006) hypothesis and with previous studies indicating that the decline in ADHD symptoms is most apparent for hyperactivity–impulsivity symptoms than in inattentiveness symptoms (Biederman et al., 2000; Fischer et al., 2005; Vaughn et al., 2011), the current study did not identify different developmental patterns for inattentiveness vs. hyperactivity-impulsivity symptoms. Although hyperactive responses showed a slower pace of change relatively to other CPT indices, they had little in common with the developmental trajectory of impulsive responses. The discrepancy from studies mentioned above may be due to the cross-sectional design of the current study that does not detect within-subjects differences. In addition, our findings may be attributed to the type of neuropsychological task used. In contrast to other CPTs, the present CPT included environmental distracters that may increase the complexity of the task, especially for ADHD children. These higher cognitive demands may explain the lack of developmental catch up which is often observed in hyperactive and impulsive responses (Biederman et al., 2000; Fischer et al., 2005; Vaughn et al., 2011).

Moreover, the majority of the behavioral studies is based on subjective measures of ADHD (e.g., parents rating, parent/children interview) and many of them included only boys (Hart et al., 1995; Biederman et al., 2000). There is evidence to suggest that when including girls in a sample, the proportion of participants with ADHD decreases with age (Cole et al., 2008). Finally, some longitudinal studies (Vaughn et al., 2011) included children who were treated by psychostimulants, whereas our sample included only drug naïve children.

It is still unclear why the difference between ADHD and non-ADHD children was more pronounced in older than in younger children. First, this finding indicates that the test provided sufficient cognitive demands for all ages, especially for older children that often find CPT too easy (Barkley, 1991; Robin, 1998; Uno et al., 2006). Second, it might also suggest that the detection of group differences may be more pronounced before adolescence than in early childhood. This finding is consistent with Drechsler et al. (2005) who found that differences between ADHD and non-ADHD children in reaction time variability and inhibitory tasks were most pronounced just before adolescence (mean age 12) than in younger children and tend to diminish into adolescence. Importantly, the increasing difference between the groups reduces the possibility of a developmental catch up before adolescence.

The findings reported here should be viewed against methodological limitations.

The most important shortcomings of this study are its relatively small sample and the imbalance of gender distribution in the younger age groups (6–7). Although CPT performance is often affected by gender (Newcorn et al., 2001; Hasson and Fine, 2012), our results consistently showed that ADHD children performed as younger typically developed children at all ages and at all CPT parameters. Therefore, differences between the two groups could not be solely attributed to differences in gender distributions. In addition, all data in this study was limited to children between 6 and 11 years. We were able to draw a behavioral curve and describe milestones of attention performance but it is yet to be uncovered which pattern characterizes later stages of development. It was also impossible to determine whether the performance of 6 years old children with ADHD resembled that of younger typically developed children.

The fact that we used cross sectional design limits the test's power to detect within-subject changes in cognitive functions. In addition, because only clinically referred children participated in the study, our results may not generalize to ADHD in the community. Furthermore, participation in the study was based on a voluntary agreement of children and their parents. This self-selected sampling strategy tends to be biased toward favoring more cooperative and motivated individuals. Therefore, it is not possible to determine whether this sample also represents other children that were not recruited and whether cooperation is confounded with ADHD variables. This limitation is typical to most clinic-based ADHD studies around the world (Lee and Ousley, 2006; Gau et al., 2010). Another limitation of the study is the exclusion of ADHD children with severe comorbidities. Since ADHD is associated with many psychiatric disorders (Gentile et al., 2006) this exclusion limits the generalization of our results. Finally, more work is needed to determine if the normalization in some ADHD symptoms reflects true remission of ADHD symptoms or is due to the developmental insensitivity of the test.

This study shed light on the age -related CPT changes in both ADHD and non-ADHD children. Our results suggest that despite improvement in CPT across childhood, ADHD continue to demonstrate impaired cognitive functioning as compared to non-ADHD children. Importantly, this study suggests that while some cognitive functions develop slower but normally, other functions (e.g., inhibitory control) do not show a clear developmental trajectory. The cross-sectional approach chosen for this study allowed frequent evaluations of typically ADHD-related behavior, which is independent upon definition of remission and persistence. Thus, it was possible to trace small and non-linear changes in performance. One of the major difficulties in early diagnosis of ADHD is that decisions about the inappropriateness of behavior in young children are based on subjective judgments of the observers (Rousseau et al., 2008; Berger and Nevo, 2011). Hence, our results highlight the importance of the CPT as an objective tool that is not affected by reporter's bias.

Future research is needed to investigate the course of ADHD symptoms in wider spectrum of age, in specific sub-types of ADHD, and in response to psychostimulants. Moreover, it is important to examine the clinical and behavioral implications of improvement in CPT performance.

## Author contributions

Itai Berger suggested the study. Itai Berger, Merav Aboud, Julia Melamed, and Hanoch Cassuto collected the data. Itai Berger, Ortal Slobodin, and Hanoch Cassuto designed the study with assistance from Merav Aboud and Julia Melamed. Ortal Slobodin, Itai Berger, and Hanoch Cassuto performed the statistical analysis. Itai Berger, Ortal Slobodin, and Hanoch Cassuto wrote the manuscript. All of the authors contributed to interpret the findings and writing the manuscript, and read and approved the final manuscript.

### Conflict of interest statement

Itai Berger serves on the scientific advisory board of Neuro-Tech Solutions Ltd. All other authors declare no conflicts of interests.

## Footnotes

^1^ The term “MOXO” derives from the world of Japanese martial arts and means a “moment of lucidity.” It refers to the moments preceding the fight, when the warrior clears his mind from distracting, unwanted thoughts, and feelings.

## Appendix A

### Description of performance indices

#### Attention

This parameter included the number of correct responses (pressing the key in response to a target stimulus), which were performed either during the stimulus presentation on the screen or during the void period that followed. Thus, it was possible to evaluate whether the participant responded correctly to the target (was attentive to the target) independently of how fast he was. Knowing how many responses are expected, it was also possible to calculate the number of times the target was presented, but the participant did not respond to it (omission errors).

#### Timing

This parameter included the number of correct responses (pressing the key in response to a target stimulus) which were performed only while the target stimulus was still presented on the screen. This parameter did not include responses that were performed during the void period (after the stimulus has disappeared).

According to the National institute of mental health (2012), inattention problems in ADHD may be expressed in “difficulties in processing information as quickly and accurately as others.” Traditionally, difficulties in timing at a CPT are evaluated by mean response time for correct responses to the target (which is interpreted as a measure of information processing and motor response speed) and by the standard deviation of response time for correct responses to the target (which is interpreted as a measure of variability or consistency) (Greenberg, 1997). In these paradigms the stimulus is presented for short and fixed periods of time and the response occurs after the stimulus has disappeared. Given the short, fixed presentation, accurate but slow participants may be mistakenly diagnosed as inattentive. While a group of patients would respond correctly if allowed more time, inattentive patients would not respond at all because they were not alert to the target. Therefore, the measurement of response time per-se, addresses only the ability to respond quickly, but not the ability to respond accurately. By implanting a void period after each stimulus and using variable presentation durations of the elements, the MOXO-CPT could distinguish accurate responses performed in “good timing” (quick and correct responses to the target performed during stimulus presentation) from accurate but slow responses (correct responses to the target performed after the stimulus presentation; during the void period). These two aspects of timing correspond to the two different problems of ADHD described by the National institute of mental health (2012); responding quickly and responding accurately.

#### Impulsivity

This parameter included the number of commission errors (responses to a non-target stimulus), performed as responses to the non-target stimuli. Usually, commission errors are coded in any case of inappropriate response to the target (e.g., pressing a random key) (Greenberg, 1997). In contrast, the MOXO-CPT's impulsivity parameter considered as impulsive behavior only the pressings on the keyboard's space–bar in response to non-target stimulus. All other non-inhibited responses (e.g., pressing the keyboard more than once) were not coded as impulsive responses (as will describe in the next paragraph).

#### Hyperactivity

This parameter included all types of commission responses that are not coded as impulsive responses. Several examples are: (1) Multiple responses- pressing the keyboard's space bar more than once (in response to target/non-target), which is commonly interpreted as a measure of motor hyper-responsivity (Greenberg, 1997). The MOXO-CPT considered as multiple responses only the second press and above (the first response would be considered as correct response with good timing, as correct response with poor timing, or as impulsive response, depends on the type of element appearing on the screen). (2) Random key pressing—pressing any keyboard button other than the space bar. By separating commission errors due to impulsive behavior from commission errors due to motor hyper-responsivity, it was possible to identify the multiple sources of response inhibition problems. Thus, the MOXO- CPT was able to differentiate impulsive responses from hyperactive responses.

## Appendix B

**Table B1:** **ADHD and control group with the minimal difference in the attention parameter, using Cohen's D measure**.

		**Control age- group**					
		**6**	**7**	**8**	**9**	**10**	**11**
ADHD age-group	6	0.60246^*^	1.07079	1.08913	1.17669	1.23039	1.21309
	7	0.09286^*^	0.72549	0.82178	1.01614	1.09397	1.17063
	8	0.50006	0.26728^*^	0.44492	0.76526	0.87376	1.09176
	9	0.25377	0.17423^*^	0.25822	0.39191	0.45764	0.51231
	10	0.51866	0.01297^*^	0.14136	0.33912	0.42792	0.53201
	11	0.83389	0.22301	0.04006^*^	0.24912	0.37256	0.58475

**Table B2:** **ADHD and control group with the minimal difference in the timing parameter, using Cohen's D measure**.

		**Control age- group**					
		**6**	**7**	**8**	**9**	**10**	**11**
ADHD age-group	6	0.44365^*^	1.09475	1.47402	1.93149	2.29619	2.55171
	7	0.09651^*^	0.55199	0.98194	1.49041	1.89167	2.22267
	8	0.61228	0.05103^*^	0.52987	1.09457	1.54204	1.95677
	9	0.86170	0.38201	0.00046^*^	0.41655	0.76452	1.02502
	10	1.15767	0.65330	0.22424^*^	0.24752	0.64407	0.96688
	11	1.53692	1.01883	0.54672	0.01755^*^	0.44408	0.85829

**Table B3:** **ADHD and control groups with the minimal difference in the hyperactivity parameter, using Cohen's D measure**.

		**Control age- group**					
		**6**	**7**	**8**	**9**	**10**	**11**
ADHD age-group	6	0.47499^*^	0.56749	0.60247	0.55383	0.62999	0.64051
	7	0.40416^*^	0.52664	0.64230	0.56272	0.72219	0.81695
	8	0.12620^*^	0.18925	0.27798	0.23131	0.33574	0.39912
	9	0.06384^*^	0.16332	0.33940	0.25653	0.45259	0.60512
	10	0.04021^*^	0.06138	0.28050	0.18594	0.41871	0.62244
	11	0.33463	0.28175	0.00765^*^	0.09309	0.15460	0.39219

**Table B4:** **ADHD and control groups with the minimal difference in the impulsivity parameter, using Cohen's D measure**.

		**Control age- group**					
		**6**	**7**	**8**	**9**	**10**	**11**
ADHD age-group	6	0.45153^*^	0.49734	0.50401	0.48621	0.55861	0.51320
	7	0.31343^*^	0.32409	0.39786	0.40298	0.57398	0.54783
	8	0.06743	0.06048^*^	0.13786	0.12947	0.30258	0.28715
	9	0.23001^*^	0.23538	0.31393	0.31876	0.49788	0.47438
	10	0.06538	0.05617^*^	0.14905	0.14761	0.35929	0.34771
	11	0.13338	0.15874	0.06945^*^	0.09495	0.11092	0.10310

* Age of control group with most resembling performance (minimal Cohen's d score).
